# HLA-Associated Immune Escape Pathways in HIV-1 Subtype B Gag, Pol and Nef Proteins

**DOI:** 10.1371/journal.pone.0006687

**Published:** 2009-08-19

**Authors:** Zabrina L. Brumme, Mina John, Jonathan M. Carlson, Chanson J. Brumme, Dennison Chan, Mark A. Brockman, Luke C. Swenson, Iris Tao, Sharon Szeto, Pamela Rosato, Jennifer Sela, Carl M. Kadie, Nicole Frahm, Christian Brander, David W. Haas, Sharon A. Riddler, Richard Haubrich, Bruce D. Walker, P. Richard Harrigan, David Heckerman, Simon Mallal

**Affiliations:** 1 Ragon Institute of MGH, MIT and Harvard, Charlestown, Massachusetts, United States of America; 2 Simon Fraser University, Burnaby, British Columbia, Canada; 3 Center for Clinical Immunology and Biomedical Statistics, Royal Perth Hospital, Murdoch University, Perth, Australia; 4 Microsoft Research, Redmond, Washington, United States of America; 5 BC Centre for Excellence in HIV/AIDS, Vancouver, British Columbia, Canada; 6 AIDS Research Institute irsiCaixa - HIVACAT, Hospital Germans Trias i Pujol, Badalona, Spain; 7 Institució Catalana de Recerca i Estudis Avançats (ICREA), Barcelona, Spain; 8 Vanderbilt University School of Medicine, Nashville, Tennessee, United States of America; 9 University of Pittsburgh, Pittsburgh, Pennsylvania, United States of America; 10 University of California San Diego, San Diego, California, United States of America; 11 Howard Hughes Medical Institute, Chevy Chase, Maryland, United States of America; 12 University of British Columbia, Vancouver, British Columbia, Canada; University of California San Francisco, United States of America

## Abstract

**Background:**

Despite the extensive genetic diversity of HIV-1, viral evolution in response to immune selective pressures follows broadly predictable mutational patterns. Sites and pathways of Human Leukocyte-Antigen (HLA)-associated polymorphisms in HIV-1 have been identified through the analysis of population-level data, but the full extent of immune escape pathways remains incompletely characterized. Here, in the largest analysis of HIV-1 subtype B sequences undertaken to date, we identify HLA-associated polymorphisms in the three HIV-1 proteins most commonly considered in cellular-based vaccine strategies. Results are organized into protein-wide escape maps illustrating the sites and pathways of HLA-driven viral evolution.

**Methodology/Principal Findings:**

HLA-associated polymorphisms were identified in HIV-1 Gag, Pol and Nef in a multicenter cohort of >1500 chronically subtype-B infected, treatment-naïve individuals from established cohorts in Canada, the USA and Western Australia. At q≤0.05, 282 codons commonly mutating under HLA-associated immune pressures were identified in these three proteins. The greatest density of associations was observed in Nef (where close to 40% of codons exhibited a significant HLA association), followed by Gag then Pol (where ∼15–20% of codons exhibited HLA associations), confirming the extensive impact of immune selection on HIV evolution and diversity. Analysis of HIV codon covariation patterns identified over 2000 codon-codon interactions at q≤0.05, illustrating the dense and complex networks of linked escape and secondary/compensatory mutations.

**Conclusions/Significance:**

The immune escape maps and associated data are intended to serve as a user-friendly guide to the locations of common escape mutations and covarying codons in HIV-1 subtype B, and as a resource facilitating the systematic identification and classification of immune escape mutations. These resources should facilitate research in HIV epitope discovery and host-pathogen co-evolution, and are relevant to the continued search for an effective CTL-based AIDS vaccine.

## Introduction

Cytotoxic T-Lymphocytes (CTL) eliminate virus-infected cells by recognizing virus-derived peptides (“epitopes”) presented by Human Leukocyte Antigen (HLA) class I molecules on the infected cell surface. The HLA-restricted CTL response is believed to play a major role in the immune control of HIV-1 infection [1], [2], [3], [4], [5], [6], and it is generally believed that an effective AIDS vaccine will have to elicit cellular as well as humoral (antibody) responses [7], [8], [9], [10], [11]. The genes encoding HLA class I are among the most polymorphic in the human genome [12]: each individual expresses up to six different class I alleles (two at each of the A, B and C loci) out of a pool of over two thousand allelic variants defined to date. Each unique HLA molecule is capable of presenting a broad but finite array of epitopes, defined by HLA allele-specific binding motifs. This extensive genetic diversity serves as a mechanism whereby the human immune system, on both the individual as well as on a population basis, is equipped to recognize a vast array of epitopes from a broad range of pathogens. In addition, this extensive diversity means that, at both the individual as well as the population level, the human immune response exerts a complex array of evolutionary selective pressures driving viral evolution [13], [14] in equally intricate, sometimes even conflicting [15], [16] ways.

One of the major mechanisms whereby HIV evades the cellular immune response is through the selection of HLA-restricted CTL escape mutations that allow the virus to evade immune recognition [17], [18], [19], [20]. Escape mutations may interfere with intracellular epitope processing [21], [22], disrupt peptide-HLA binding [23], [24], or disrupt recognition of the peptide/HLA complex by the T-cell receptor [25], [26].

Despite the extensive genetic diversity of both HIV-1 and HLA, recent studies indicate that viral evolution in response to immune selective pressures follows generally predictable patterns and kinetics [13], [14], [15], [27], [28], [29], [30]. For example, in B*57-expressing individuals, the B*57-associated T242N escape mutation in Gag is selected mere weeks after infection [31], [32], [33], whereas the B*27-associated R264K (Gag) may take years to develop despite strong continuous immune pressure in individuals expressing B*27 [24], [34]. Furthermore, both T242N and R264K are typically accompanied by a well-defined set of compensatory mutations [24], [35], [36]. The development of improved statistical methods [37] combined with the availability of large cohorts for which HIV sequences and HLA data are available has facilitated the systematic identification of HLA-associated CTL escape mutations, both within [15], [28], [29], [38] and across [27] HIV subtypes. Due to the extensive diversity of both HLA and HIV, the identification of mutational escape patterns requires large, well-powered datasets; thus, additional data are needed in order to refine existing escape maps. Indeed, just as the systematic identification of antiretroviral resistance mutations [39] has been of paramount importance to the design and monitoring of HIV therapies [40], the comprehensive elucidation of immune escape pathways will be of relevance to HIV vaccine research.

Here, we identify HLA-associated polymorphisms within the three HIV-1 proteins most commonly considered in cellular-based vaccine design strategies (Gag, Pol and Nef) in a combined analysis of three established cohorts totaling >1500 HIV-infected, antiretroviral-naïve individuals. We organize results into protein-wide escape maps illustrating the sites and pathways of immune-driven viral evolution, and hope that these maps will serve as useful reference material for researchers interested in CTL epitope discovery, host-pathogen co-evolution, and HIV vaccine design.

## Materials and Methods

### Analysis of three established cohorts and formation of the International HIV Adaptation Collaborative (IHAC)

We merged HLA class I and HIV-1 Gag, Pol and Nef sequence data from three existing cohorts of chronically-HIV-infected, antiretroviral naïve individuals previously featured in population-level investigations of HIV immune escape: the British Columbia HOMER cohort (British Columbia, Canada, N = 765) [15], [38], the Western Australian HIV Cohort Study (WAHCS; Western Australia, Australia N = 230) [14], [37], and US AIDS Clinical Trials Group (ACTG) protocols 5142 participants [41] who also provided human DNA under ACTG protocol 5128 [42] (N = 555). We have assigned the name “International HIV Adaptation Collaborative” (IHAC) to describe this multicenter, international cohort.

### Ethics Statement

Ethical Approval was obtained through the following Institutional Review Boards: Providence Health Care/University of British Columbia; Royal Perth Hospital Ethics Committee; and the NIH's National Institute of Allergy and Infectious Diseases (NIAID) Clinical Science Review Committee (CSRC).

### Genotyping methods and inter-laboratory methods comparison for quality control

HIV and HLA data collection for HOMER cohort participants was performed at the BC Centre for Excellence in HIV/AIDS, Vancouver, Canada. Here, HIV RNA was extracted from plasma using standard methods and regions of interest amplified by nested RT-PCR using HIV-specific primers. PCR amplicons were bulk sequenced on an Applied Biosystems 3100, 3700 and/or 3730 automated DNA sequencer. Data were analyzed using ‘Sequencher’ (Genecodes) or custom software RE_Call. Nucleotide mixtures were called if the height of the secondary peak exceeded 25% of the height of the dominant peak. HLA class I typing was performed using an in-house sequence-based typing protocol and interpretation algorithm [15].

HIV and HLA data collection for the WAHCS and ACTG 5142/5128 cohort participants was performed at the Centre for Clinical Immunology and Biological Statistics (CCIBS) laboratory in Perth, Australia. Plasma HIV RNA was extracted using standard methods and nearly complete viral genomes amplified using nested RT-PCR. PCR amplicons were bulk-sequenced using Applied Biosystems 3730 automated sequencing. Data were analyzed using semi-automated ASSIGN software with a nucleotide mixture threshold of 15% after consideration of the signal/noise ratio, yielding near-full genome sequences. High-resolution HLA class I typing was performed using sequence-based methods and allele interpretation was performed using ASSIGN [41].

To rule out potential biases due to differences in sequence analysis strategies between study sites, an inter-laboratory DNA sequencing comparison between the Vancouver and Perth laboratories was performed prior to merging data. A total of 42599 base pairs of sequence data covering Gag, Pol and Nef were exchanged and analyzed using site-specific software and procedures in a blinded fashion. Overall inter-laboratory concordance was 42429 out of 42599 calls, or 99.6%. Of the 170 discordant calls, 169 (99.4%) were due to the presence of a nucleotide mixture called by one laboratory but not the other, with a tendency of the Vancouver lab to call more mixtures than the Perth lab.

After verification of inter-laboratory concordance, Gag, Pol and Nef sequences were extracted from the nearly-full genome WAHCS/ACTG sequences using GeneCutter (http://www.hiv.lanl.gov/content/sequence/GENE_CUTTER/cutter.html) before merging with the BC HOMER sequence data. The merged sequence datasets were aligned to HIV-1 subtype B reference strain HXB2 (GenBank Accession No. K03455) using a modified NAP algorithm [40]. HLA class I types were summarized to two-digit resolution. Final HLA/HIV sequence datasets comprised N = 1294, 1383 and 1299 for Gag, Pol and Nef, respectively. Sequence subtypes verified by comparison to subtype references in the Los Alamos HIV Database (http://www.hiv.lanl.gov); >95% of sequences in this study were subtype B.

### GenBank Accession Numbers

Gag, protease/RT (codons 1–400 only) and nef sequences from the HOMER cohort were previously deposited in GenBank [15], [38]. Accession numbers of additional HOMER protease/RT (codon 1–400 only) sequences included in the present study are GQ303719-GQ303727; full-length protease/RT sequences are GQ303728-GQ303867 and HOMER RT codon 401–560 sequences are GQ303868-GQ304249. HOMER integrase sequences are FJ812899-FJ813480. Linked HLA/HIV datasets from the BC HOMER cohort are available for sharing with individual researchers following application to, and approval by the UBC/Providence Health Care Research Ethics Board; please contact the corresponding author for more information. GenBank Accession Numbers for ACTG 5142/5128 cohort sequences are GQ371216-GQ371763 (Gag), GQ371764-GQ372317 (Pol) and GQ372318-GQ372824 plus GQ398382-GQ398387 (Nef). GenBank Accession numbers for the full/partial HIV genome sequences from WAHCS, from which Gag, Pol and Nef were extracted and used here, are AY856956-AY857186.

### Identification of HLA-associated polymorphisms: Overview

The identification of HLA-associated polymorphisms in population-based datasets is complicated by three potential confounding factors: HIV phylogeny, HIV codon covariation, and Linkage Disequilibrium between HLA alleles [27]. HIV phylogeny acts as a confounder because HIV sequences are related to one another through descent from a common ancestor, with sequences displaying greater or lesser similarity to one another depending on the length of time since divergence. Thus, statistical tests that assume independent and identically distributed (iid) observations, such as chi-squared or Fisher's exact tests, may lead to inflated false-negative and false-positive rates if applied directly [27], [37]. Similarly, Linkage Disequilibrium (LD) between HLA alleles also exerts confounding effects. If LD is not addressed, linked HLA alleles may appear associated with the same mutational patterns, when in reality, escape is driven by one allele only [14], [15], [27], [28], [29]. For this reason, analytical methods have been developed to account for both HIV phylogeny and HLA LD [27].

HIV codon covariation acts as an additional, albeit more subtle, confounder [27]. Although the phylogenetic tree adjusts for the underlying evolutionary relationships between HIV sequences, immune selection pressures may lead to reproducible patterns of mutations at linked sites, even in sequences located far apart in the tree. An example of this may be an escape pattern where a primary mutation is first selected in context of a specific HLA allele, followed by a compensatory mutation at a secondary site [24], [35], [36]. If codon covariation is not accounted for, both the primary and secondary sites may be identified as being associated with the HLA allele in question. Technically, this result would not be incorrect (as both primary and secondary mutations are selected by the HLA allele). However, if the goal is to discriminate between HLA-associated polymorphisms selected directly (for example, mutations that compromise epitope processing, peptide-HLA binding and/or T-cell recognition), from those that are selected indirectly (such as compensatory or secondary mutations), correction for codon covariation is necessary. A method that simultaneously accounts for HIV phylogeny and codon covariation, in addition to HLA linkage disequilibrium, has recently been developed by Carlson et al [27], and we have applied it in this study.

### Identification of HLA-associated polymorphisms: statistical methods

A detailed description of the HIV Phylogeny, HLA LD and HIV codon-covariation-corrected method is published in [27]. Briefly, a maximum likelihood phylogenetic tree is constructed for each gene and a model of conditional adaptation is inferred for each observed amino acid at each codon. In this model, the amino acid is assumed to evolve independently down the phylogeny, until it reaches the observed hosts. In each host, the selection pressure arising from HLA-mediated T-cell responses and amino acid covariation is directly modeled using a stochastic additive process. To identify which factors contribute to the selection pressure, a forward selection procedure is employed, in which the most significant association is iteratively added to the model, with p-values computed using the likelihood ratio test. To increase our statistical power, each codon is divided into a set of binary variables, one for each observed amino acid. In addition, we only consider pairs of variables for which the observed or expected value for each value of the contingency table is at least three.

### Definition of Statistical Significance

Statistical significance is reported using q-values, the p-value analogue of the false discovery rate (FDR) for each p-value threshold [43]. The FDR is the expected proportion of false positives among results deemed significant at a given threshold. For example, at a q≤0.2, we expect a false-positive proportion of 20% among identified associations. The q-value threshold used for constructing the immune escape maps was q≤0.05, meaning that we would expect only a 5% false-positive proportion among associations displayed on our maps. Tables S1 and S2 list all HLA- and covariation associations, respectively, with q≤0.2 (see supporting information).

### Classification and Nomenclature of HLA-associated polymorphisms

Using this method, HLA-associated polymorphisms are grouped into two categories: (1) amino acids significantly *enriched* in the presence of the HLA allele in question (and vice versa), and (2) amino acids significantly *depleted* in the presence of the HLA allele in question (and vice versa). We refer to these two categories as the “adapted” and “nonadapted” forms, respectively. Previous studies, including some by our group, have employed various nomenclature systems for these polymorphisms: the “adapted” forms may also be referred to as “escape mutants” or “resistant forms”, while the “nonadapted” forms have been referred to as “susceptible”, “wild-type” and/or “reversion” forms. We will endeavour to use the “adapted/nonadapted” nomenclature in all future studies of this type.

### HIV covariation-corrected analysis: a point to consider

HIV proteins often contain multiple epitopes restricted by the same HLA allele (B*57 TW10 and IW9 in Gag, for example). On occasion, we have observed that the covariation-corrected analysis identifies mutations in epitopes restricted by the same HLA allele as being “linked”, when in fact a more likely explanation is that that they arise due to HLA-restricted targeting and escape within multiple epitopes, either simultaneously or sequentially over the disease course [27], [32], [44], [45], [46], [47]. In order to not exclude any potentially important escape mutations from our figures, we ran the analysis with and without the covariation correction, and included all HLA-associated polymorphisms identified by either method in the escape maps. In the escape maps, the covariation-corrected and uncorrected associations are differentiated by the use of uppercase and lowercase letters, respectively.

## Results

HLA-associated polymorphisms were identified in HIV-1 Gag, Pol and Nef in a multicenter cohort of >1500 chronically-infected, treatment-naïve individuals using published methods featuring a correction for HIV phylogeny, HLA linkage disequilibrium and HIV codon covariation [27]. The false discovery rate [43] was used to account for multiple tests.

At the conservative threshold of q≤0.05, 282 HIV codons commonly mutating under HLA-associated immune pressure in Gag, Pol and Nef were identified. These polymorphisms were observed at 74 (of 206; 36%) Nef codons, 80 (of 500; 16%) Gag codons, and 128 (of 947; 14%) codons in Pol. At a more liberal threshold of q≤0.2, the total number of observed codons harboring HLA-associated polymorphisms increased to 442, which included 113 (55%), 130 (26%), 199 (21%) of codons in Nef, Gag and Pol, respectively. These data confirm the results of previous population-based studies reporting greater density of HLA-associated polymorphisms in Nef than in Gag or Pol [15], [29], [41], [48]. Moreover results underscore the observation that the effects of HLA-associated selection pressures on HIV-1 evolution are extensive and predictable.

**Figure 1:**
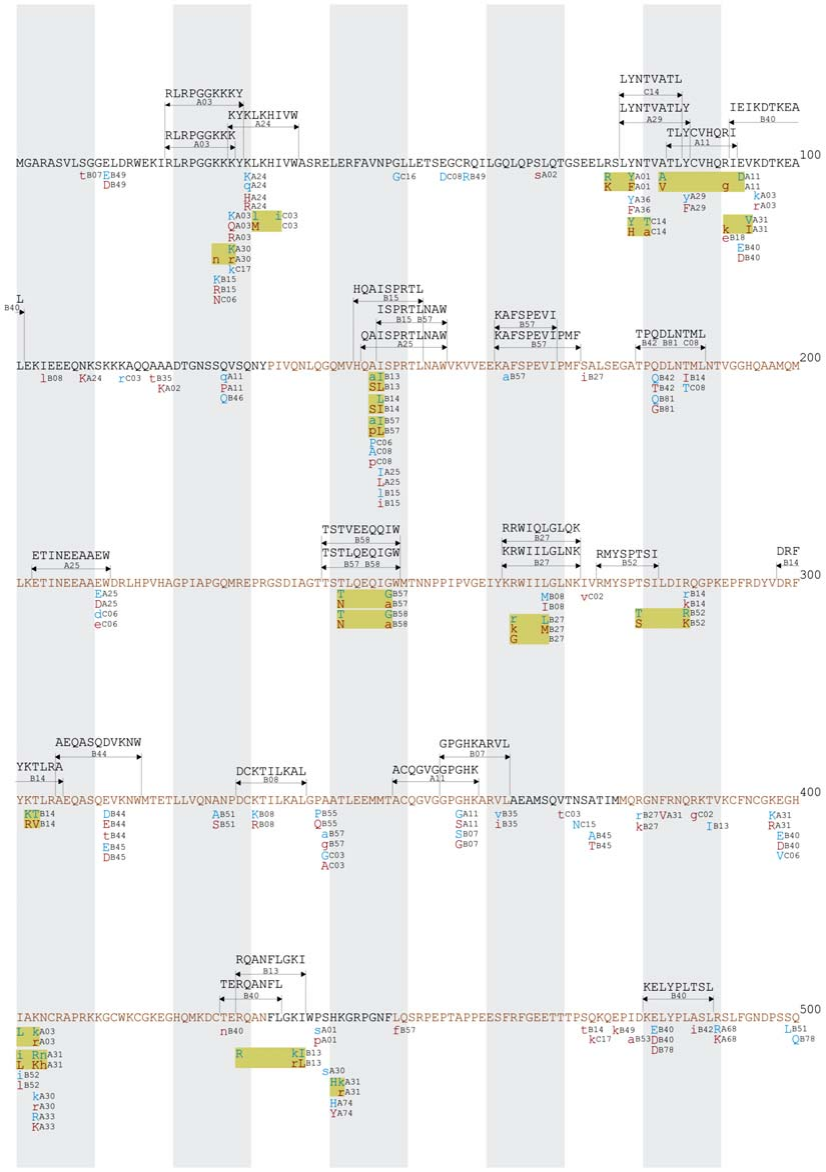
Gag Immune Escape Map. Escape maps indicate the locations, specific residues and HLA restrictions of HLA-associated polymorphisms. The HIV-1 consensus B amino acid sequence is used as a reference. Alternating black and brown letters in the consensus amino acid sequence distinguish the different proteins in HIV-1 Gag (p17, p24, p2, p7, p1, p6). One hundred amino acids are displayed per line. Shaded vertical bars separate blocks of 10 amino acids. “Adapted” amino acids (those *enriched* in the presence of the HLA allele) are red. “Non-adapted” amino acids (those *depleted* in the presence of the HLA allele) are blue. UPPERCASE letters distinguish polymorphisms that survive correction for HIV codon covariation (“direct” associations), while lowercase letters distinguish polymorphisms that do not survive correction for codon covariation (“indirect” associations). Polymorphisms associated with the same HLA allele that occur in proximity to one another are grouped together in yellow boxes. Optimally-defined CTL epitopes containing HLA-associated polymorphisms are indicated above the consensus sequence. *Note that the escape map does not list the locations of all published CTL epitopes. This is available at* http://www.hiv.lanl.gov/content/immunology. The escape maps show all HLA-associated polymorphisms with q≤0.05. A complete listing of all HLA-associated polymorphisms with q≤0.2 is provided in Table S1.

**Figure 2 pone-0006687-g002:**
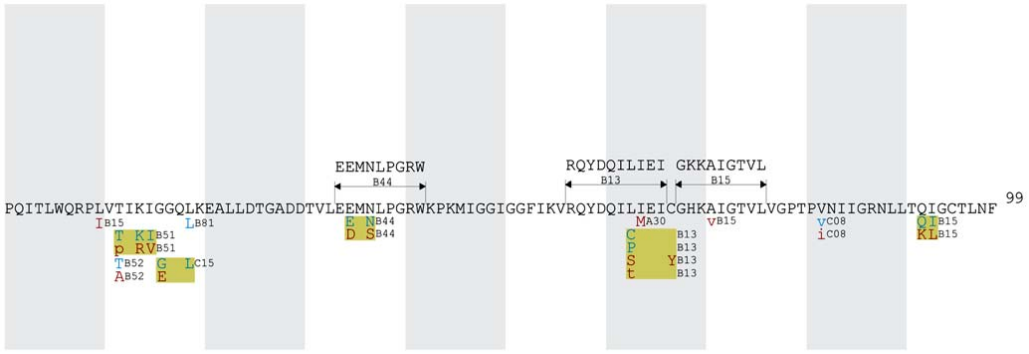
Protease Immune Escape Map.

**Figure 3 pone-0006687-g003:**
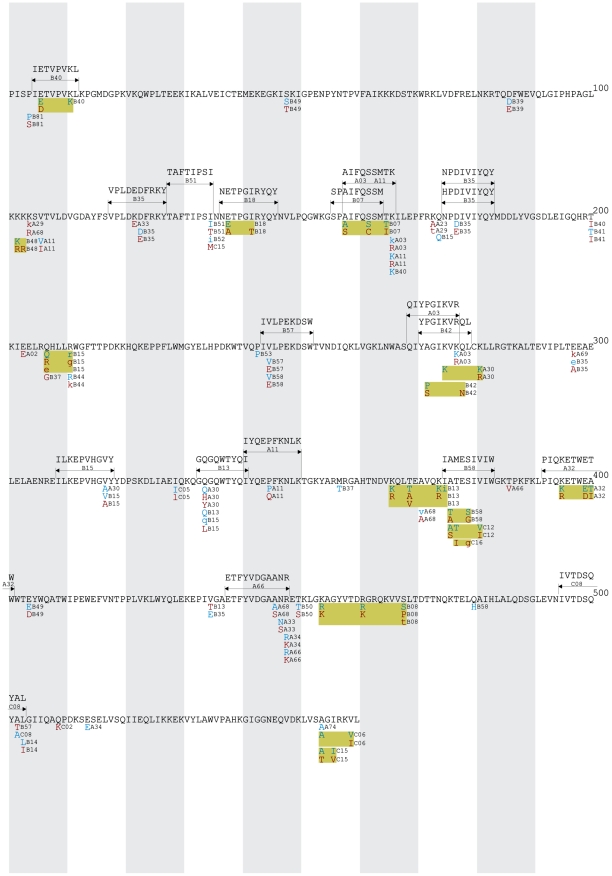
Reverse Transcriptase Immune Escape Map.

**Figure 4 pone-0006687-g004:**
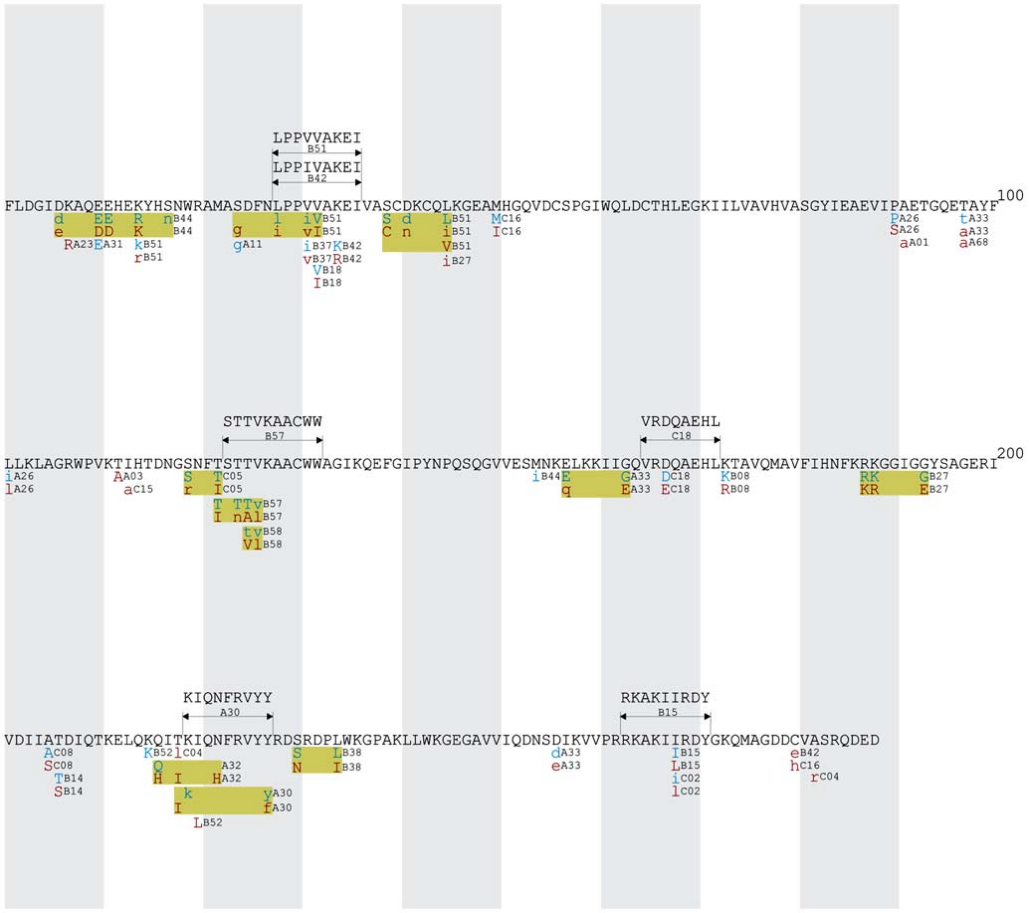
Integrase Immune Escape Map.

**Figure 5:**
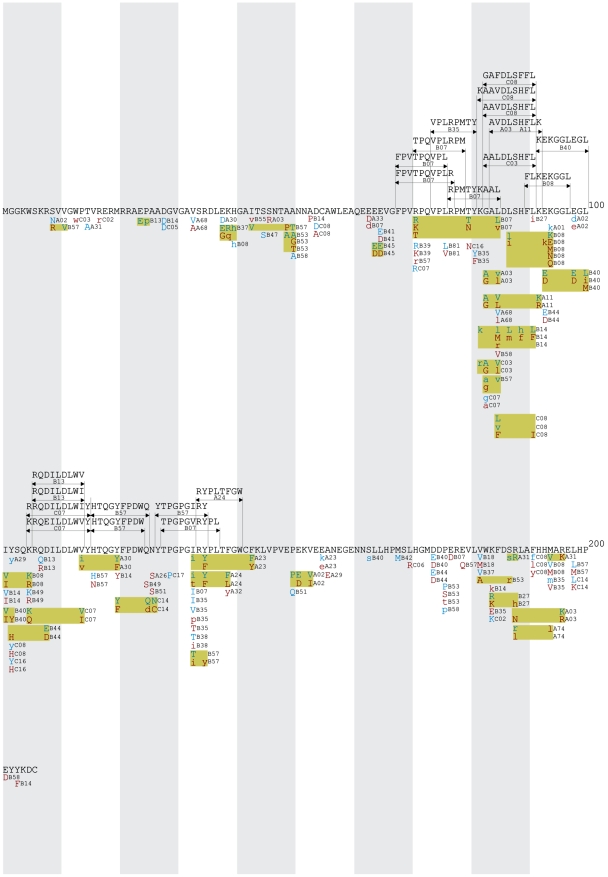
Nef Immune Escape Map.

All HLA-associated polymorphisms at q≤0.05 were organized into gene-wide “immune escape maps” (Figures 1, 2, 3, 4, 5) indicating their location, HLA restriction, specific amino acids, and their direction of association (“adapted” vs. “non-adapted”) with respect to the current HIV subtype B consensus sequence (http://www.hiv.lanl.gov). Published, optimally-described CTL epitopes [49] containing HLA-associated polymorphisms are also shown. In addition, the maps discriminate between HLA-associated polymorphisms directly attributable to selection pressure by the allele (meaning that they survive correction for HIV covariation), from those that may be better explained indirectly (meaning that their occurrence may be better explained by the presence of an HLA-associated covarying residue, rather than the allele itself). The full list of direct (covariation-corrected) plus indirect (covariation uncorrected) HLA-associated polymorphisms within each viral protein at q≤0.2 is provided in Table S1.

As described in the methods, we also undertook an HIV codon-covariation analysis that, besides identifying direct HLA-associated polymorphisms, also identified all pairwise amino acid-amino acid (aa-aa) associations within a given HIV protein [27]. The HIV codon covariation analysis can be used to identify linked pathways of immune escape, as well as putative secondary and/or compensatory mutations associated with a primary escape site. The codon covariation analysis identified >7000 intra-protein aa-aa correlations occurring at >4500 codon pairs, illustrating the dense and complex networks of covarying amino acids in HIV (Table S2). Indeed, if one sums up the total number of codons harboring HLA-associated polymorphisms, plus the co-varying sites immediately associated with them, the total proportion of codons in Nef that are either directly or indirectly associated with HLA selection pressures reaches 77%. For Gag and Pol, the corresponding proportions are 55% and 44%, respectively.

**Figure 6:**
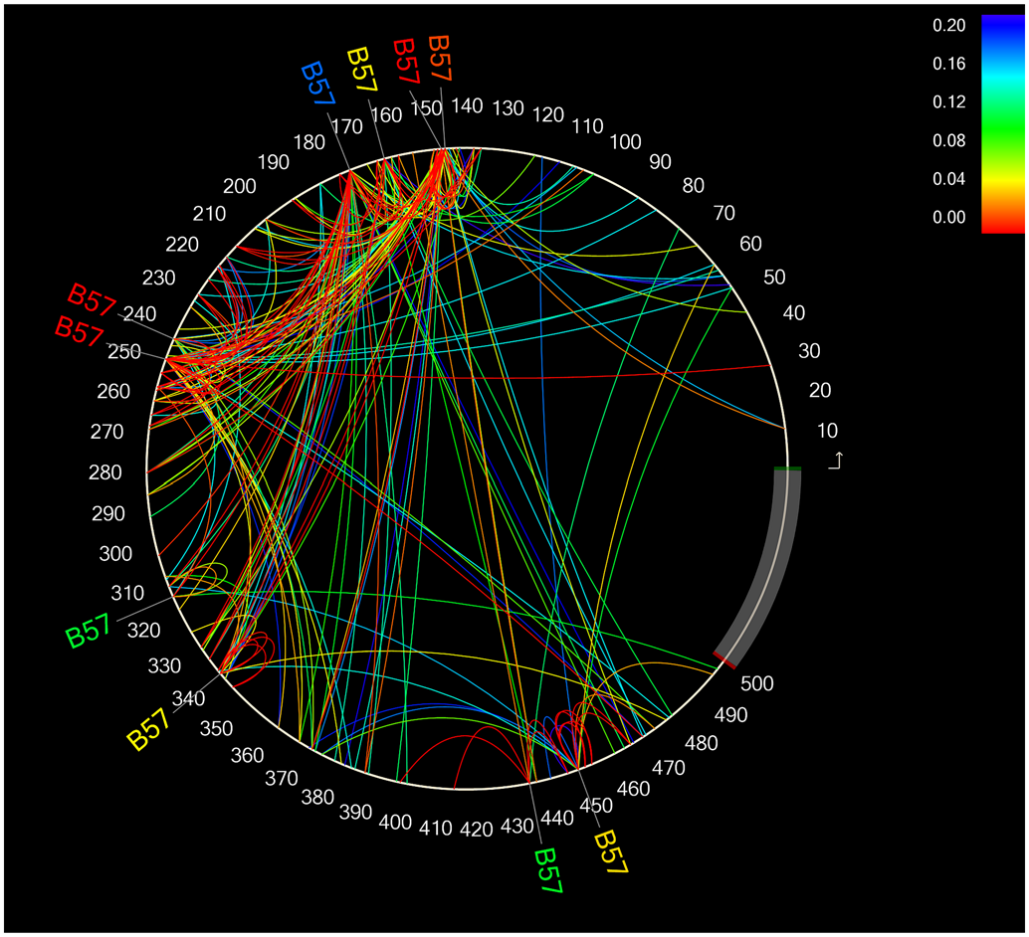
HLA-B*57-associated escape and covariation pathways in HIV-1 Gag. The 500 amino acids of consensus B Gag are drawn counterclockwise, with the N-terminus of Gag at the 3 o'clock position. All direct (covariation-corrected) and indirect (covariation uncorrected) B*57-associated polymorphisms at q≤0.2 are identified at their respective positions along the circle's circumference, while covarying amino acids (also q≤0.2) are joined together by arcs within the circle. Note that this figure is limited to “one-hop” covarying amino acids only, meaning that only the codons directly associated with variation at a B*57-associated sites are shown. (Our analyses also identify, for example, codons associated with variation at the “one-hop” sites, and so on and so forth, but for simplicity we have limited the figure to the “one-hop” sites only. The strength of the association between two covarying codons (expressed in terms of q-value) is indicated by the color of the arc. The program used to construct these figures is available at http://research.microsoft.com/en-us/um/redmond/projects/MSCompBio/PhyloDViewer/ [27].

**Figure 7:**
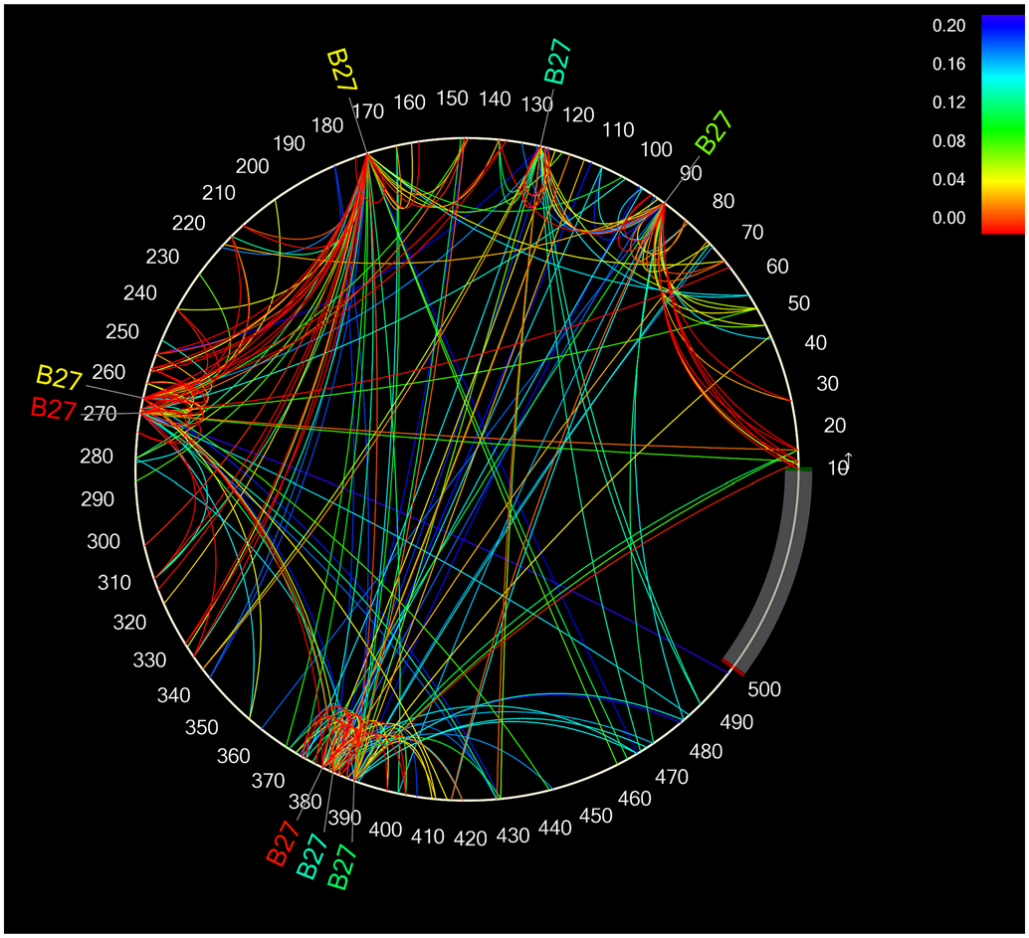
HLA-B*27-associated escape and covariation pathways in HIV-1 Gag.

The sheer density of the intraprotein codon covariation network renders the task of displaying these data rather challenging, but Carlson *et al* have developed an elegant tool for data visualization that is freely available [27]. Here, the amino acid sequence of a protein is displayed in a counterclockwise circle starting at the 3 o'clock position (Figures 6 and 7). Any HLA alleles associated with variation at those sites are indicated at the corresponding positions outside the circle, while covarying amino acids are joined together by arcs within the circle. The strength of the association (q-value) is indicated by the color of the arc.

HLA-associated intraprotein codon networks in Gag for HLA-B*57 and HLA-B*27 are shown in Figures 6 and 7, respectively. These two alleles were chosen as examples due to their association with slower HIV disease progression in numerous epidemiologic studies [5], [34], [50]. Similarly, Gag was chosen in light of accumulating evidence that Gag-specific CD8 T-cell responses may contribute substantially to HIV immune containment [51], [52], [53] as well as the observation that B*57 and B*27-associated escape mutations in Gag are associated with measurable costs to viral replication capacity [35], [36], [54], which may be partially rescued by compensatory mutations at secondary sites [35], [36]. All direct (covariation-corrected) and indirect (covariation uncorrected) B*57-associated Gag polymorphisms at q≤0.2 are identified at their respective positions along the circle's circumference: For B*57 (figure 6), this corresponds to codons 146, 147, 163, 173, 242, 248, 315, 340, 435 and 449. Within the circle, all q≤0.2 “one-hop” associations with these codons (meaning, Gag codons identified as covarying with them) are connected via arcs. For example, if the B*57-associated polymorphism at Gag position 242 is considered the “predictor variable” (see Table S2), then the residues positively associated with it (“adapted” associations) and/or negatively associated with it (“nonadapted” associations) are located at codons 146, 147, 215, 228, 230, 241, 243, 248, 256, 310, 340 and 373. If position 242 is considered the “target variable”, then the covarying residues positively and negatively associated with it are located at codons 109, 219, 292, 373, 469 and 473. It is important to note in the case of aa-aa associations, the use of “predictor” and “target” terminology should not be interpreted as suggesting a directional association between these polymorphisms or a specific temporal order of selection; rather, it is more appropriate to simply interpret these as codon-codon pairs. Therefore, if one is interested using Table S2 to look up all codons positively and/or negatively associated with Gag codon 242, one should investigate all “target” codons that appear when 242 is set as the “predictor” variable, *and vice versa*. The union of these two queries will provide a list of specific codons and residues that are positively and/or negatively associated with variation at codon 242.

Note that our analysis also identifies “two-hop” associations (meaning, codons that positively and/or negatively covary with the “one-hop” sites), however these are not shown on the figure due to the high density of the resulting networks. The full list of intraprotein covarying codons is provided in Table S2.

## Discussion

HLA-associated polymorphisms were identified in HIV-1 Gag, Pol and Nef in a combined cohort of >1500 chronically-infected, treatment-naïve individuals from established cohorts in Canada, the USA and Western Australia. These cohorts have previously been independently investigated for HLA-associated polymorphisms; however by merging the data and re-analyzing as a whole, we achieved the highest-powered dataset to date to identify HLA associations in HIV subtype B. Indeed, where previous studies had employed a significance threshold of q≤0.2 when reporting associations, here we have lowered the threshold to q≤0.05, thus focusing on sites with the strongest statistical support for HLA-driven adaptation.

The current immune escape maps incorporate some improvements over previous iterations. Firstly, the maps cover all proteins in Pol (including RNAseH and Integrase), instead of just protease/RT as in previous studies [15]. Secondly, all associations, regardless of proximity to known epitopes, are displayed on a single map so that escape patterns in a protein can be visualized globally. Note that, in the case where an HLA-associated polymorphism does not fall within a known optimally-described epitope, we have not attempted to predict the likeliest epitope as has been done previously. This was done in order to avoid forcing an epitope prediction in the case where the HLA association may be attributable to another mechanism (for example a processing escape mutation occurring distant from a published epitope), and also to avoid favoring a particular epitope prediction algorithm among the many that are available (e.g.: MotifScan http://www.hiv.lanl.gov/content/immunology/motif_scan; Epipred http://atom.research.microsoft.com/bio/epipred.aspx [55]; SYFPEITHI http://www.syfpeithi.de/Scripts/MHCServer.dll/EpitopePrediction.htm, [56], NetCTL 1.2 http://www.cbs.dtu.dk/services/NetCTL/ [57], and various others [58], [59]). That being said, visual inspection of the maps reveals strong evidence for the existence of a number of novel CTL epitopes, particularly in Pol where epitope mapping initiatives may not have been as exhaustive compared to Gag and Nef. Thirdly, the incorporation of a multivariate correction for HLA linkage disequilibrium allows the identification of the HLA allele directly responsible for the association, rather than the manual assignment of the responsible allele using p-values post-hoc as employed in previous studies [15]. Finally, the incorporation of a multivariate correction for codon covariation represents an important step forward [27]. It allows us to discriminate HIV polymorphisms directly attributable to selection pressure by the HLA allele in question, from those who may be better explained indirectly (meaning that their occurrence may be better explained by the presence of an HLA-associated covarying residue, rather than possession of the allele itself). In addition, it allows us to comprehensively identify positively and negatively covarying amino acids across proteins (Table S2), thus providing candidate lists for secondary and/or compensatory mutations associated with known escape sites. Indeed, Carlson *et al*. [27] demonstrated that the codon covariation analysis accurately re-capitulates known pathways of B*57 and B*27-associated escape in Gag [35], [36], [60], supporting the use of this tool for the identification of secondary escape patterns for additional HLA-associated escape mutations.

Just as the standardized identification of drug resistance mutations [39] has been essential to both basic research as well as the clinical monitoring of HIV-infected individuals, we hope that the identification of immune escape pathways will be equally relevant to HIV immunology/virology research and AIDS vaccine design. Our results confirm the strong influence of immune escape on HIV diversity [13], [14], but more importantly underscore the reproducibility and predictability of immune escape in response to specific HLA pressures [15], [27], [28], [29], [38]. We hope these maps and tables will be useful to those interested in CTL epitope discovery, the effects of escape and compensatory mutations on viral replication and pathogenesis, the design of novel vaccines, as well as the broader question of host-pathogen co-evolution. Finally, we have assigned the name “International HIV Adaptation Collaborative” (IHAC) to describe the current multicenter cohort with the hope that this initiative may be expanded to include additional cohorts worldwide in the future. In particular, the merging of data and cohorts across different HIV-1 subtypes [27] will allow us to further explore similarities and differences in HLA-driven polymorphism patterns across subtypes.

## Supporting Information

Table S1 HLA-associated polymorphisms in HIV-1 Gag, Protease, Reverse Transcriptase, Integrase and Nef (all q<0.2). Consistent with the immune escape maps, amino acid numbering begins with 1 for each individual protein (where individual proteins are defined as Gag, Protease, Reverse Transcriptase, Integrase and Nef). Also consistent with the escape maps, the direction of the association (adapted vs. nonadapted) for the specific HLA in question is differentiated by red and blue lettering, respectively. Direct (covariation-corrected) and indirect (non-covariation corrected) are also differentiated. P- and q-values represent the minimum values observed in the covariation corrected and non-covariation corrected analyses.
(0.23 MB XLS)

Table S2 Amino acid-amino acid (aa-aa) associations in HIV-1 Gag, Protease, Reverse Transcriptase, Integrase and Nef (all q<0.2). Consistent with the immune escape maps and supplementary table 1, amino acid numbering begins with 1 for each individual protein. Predictor Codon and Target Codon refer to the predictor and target attributes, respectively, however it is important to note that this terminology does not imply a specific direction of association. For example, if one is interested in all codons that covary with Gag codon 242, one should investigate all target codons that appear when 242 is set as the predictor variable, and vice versa. The union of these two searches will provide a list of candidate codons that covary with codon 242. In the case of aa-aa associations, adapted refers to positive associations (ie amino acid pairs that statistically tend to co-exist/co-vary) while nonadapted identifies negative associations (ie amino acids that statistically tend not to be found together). Finally, note that in the original analysis, HIV codon covariation was analyzed across all three Pol proteins simultaneously. However, to maintain consistency with Figures 1-5 in the paper, the data listed in this supplementary table are limited to intra-protein associations only, a fact which should be considered when interpreting the q-values for aa-aa associations in Protease, RT and Integrase.
(1.13 MB XLS)
